# Exploring Morphology of Thermoplasmonic Nanoparticles to Synergize Immunotherapeutic Fibroblast Activation Protein‐Positive Cell Sensitization and Photothermal Therapy

**DOI:** 10.1002/smsc.202500099

**Published:** 2025-05-26

**Authors:** Ahmed Alsadig, Xuan Peng, Hugo Boutier, Liliana R. Loureiro, Anja Feldmann, René Hübner, Humberto Cabrera, Manja Kubeil, Michael Bachmann, Larysa Baraban

**Affiliations:** ^1^ Institute of Radiopharmaceutical Cancer Research Helmholtz‐Zentrum Dresden‐Rossendorf e. V. 01328 Dresden Germany; ^2^ Institute of Ion Beam Physics and Materials Research Helmholtz‐Zenztrum Dresden‐Rossendorf e. V. 01328 Dresden Germany; ^3^ The Abdus Salam International Centre for Theoretical Physics MLab STI Unit 34151 Trieste Italy; ^4^ PDMFC Rua Fradesso da Silveira 4‐1B Lisboa 1300‐609 Portugal; ^5^ Institute of Resource Ecology Helmholtz‐Zentrum Dresden‐Ressensorf e. V. 01328 Dresden Germany; ^6^ Else Kröner Fresenius Center for Digital Health Faculty of Medicine Carl Gustav Carus Technische Universität Dresden 01307 Dresden Germany

**Keywords:** fibroblast activation protein, gold nanoparticles, immunotherapeutic target modules, photothermal therapy, specific cell targeting, thermal lens spectroscopy

## Abstract

The precision of photothermal therapy (PTT) is often hindered by the challenge of achieving selective delivery of thermoplasmonic nanostructures to tumors. Active targeting, which leverages synthetic molecular complexes to address receptors overexpressed by malignant cells, enables such specificity and facilitates the combination of the PTT with other anticancer therapies. In this study, we developed thermoplasmonic nanoconjugates consisting of (i) 20 nm spherical gold nanoparticles (AuNPs) or gold nanostars (AuNSs) as nanocarriers, and (ii) surface‐passivated antibody‐based fibroblast activation protein (FAP)‐targeting modules, used in adaptive chimeric antigen receptor T‐cells immunotherapy. The nanoconjugates demonstrated excellent stability and specific binding to FAP‐expressing fibrosarcoma HT1080 genetically modified to express human FAP, as confirmed by fluorescence activated cell sorting, immunofluorescence, and surface plasmon resonance scattering imaging. Moreover, the nanocarriers showed significant photothermal conversion after visible and near‐infrared irradiation. Quantitative thermal lens spectroscopy demonstrated the superior photothermal capability of AuNSs, achieving up to 1.5‐fold greater thermal enhancement than AuNPs under identical conditions. This synergistic approach, combining targeted immunotherapy with the thermoplasmonic nanocarriers, not only streamlines nanoparticle delivery, increasing photothermal yield and therapeutic efficacy but also offers a comprehensive and potent strategy for cancer treatment with the potential for superior outcomes across multiple modalities.

## Graphical abstract

Schematic of spherical and branched gold nanoparticle‐based nanocarriers conjugated with targeting modules (TMs), previously designed for chimeric antigen receptor T‐cell (CAR T‐cell) recognition of fibroblast activation protein (FAP). By combining immunotherapeutic TMs with thermoplasmonic AuNPs, these nanoconjugates selectively sensitize FAP‐expressing cells to hyperthermia, offering a promising platform for solid tumor‐targeted theranostics.

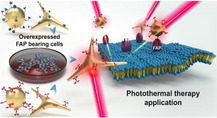

## 1 Introduction

**Scheme 1:**
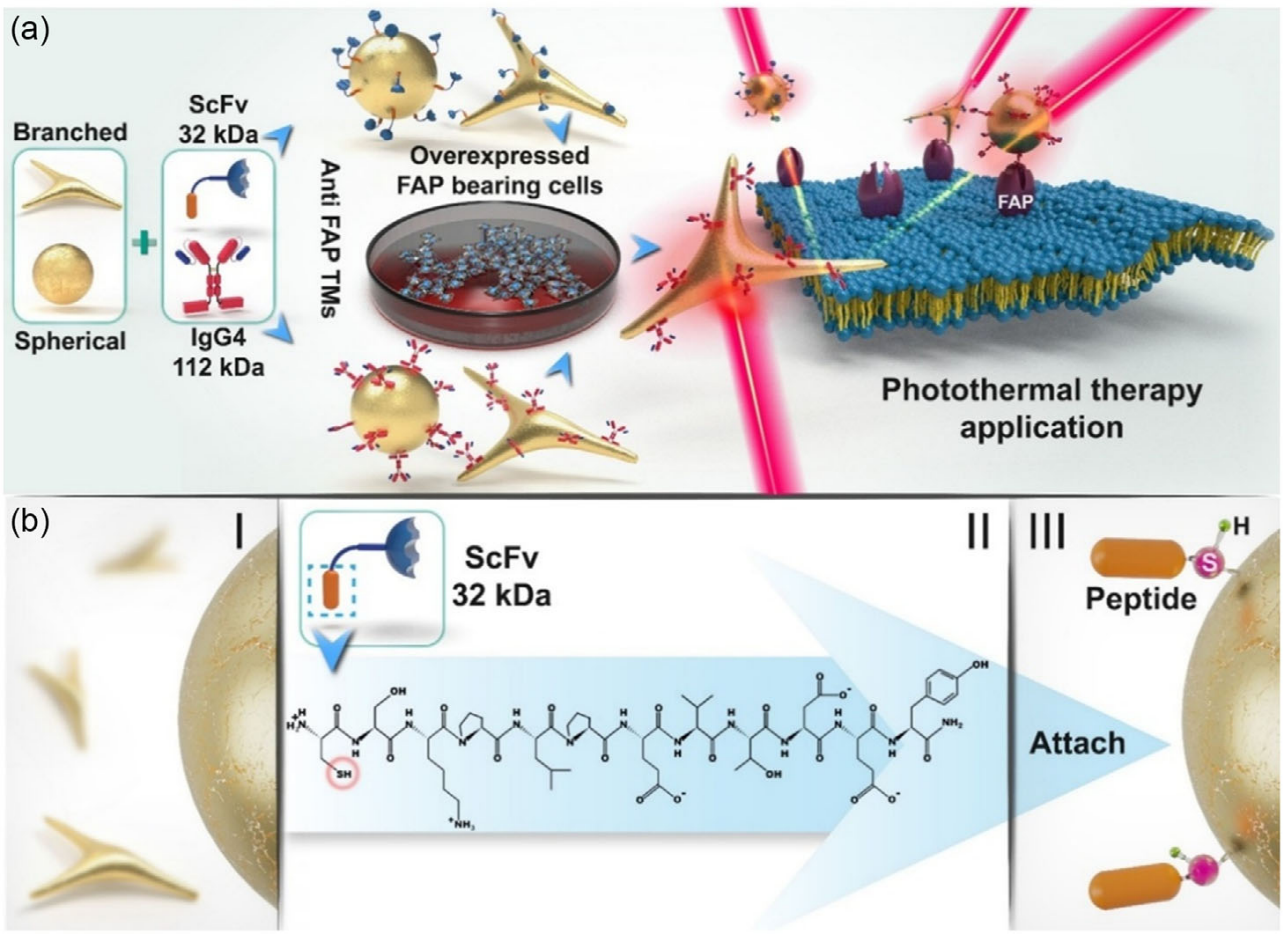
a) Schematic illustration of the Au‐nanoplatforms using both spherical and branched AuNPs as nanocarriers for anti‐FAP TMs. The blue TM component is the ScFv that specifically binds to FAP and is directed toward the peptide epitope E5B9, which interacts with AuNP surfaces. The red TM component consists of human IgG (IgG4). This versatile approach aims to enhance cell labeling of FAP‐overexpressing cancer cells, enabling their subsequent ablation via photothermal conversion. b) Surface biofunctionalization of AuNPs was achieved through the Au‐S covalent bond between the cysteine‐terminated E5B9 peptide epitope (shown in orange with sulfur atom acts as the anchor point as shown in pink), and Au surfaces (I–III), facilitating the formation of a protein monolayer.

Plasmonic nanometals exhibit fascinating optical phenomena such as local field enhancement, hot electrons generations, and photothermal conversion, all arising from quantized surface electron oscillations.^[^ ^1^ ^]^ These materials are considered particularly promising as their structural and dimensional alterations allow for precise tuning of their surface plasmon resonances (SPR), opening up prospects for controlling light‐matter interactions.^[^ ^2^, ^3^ ^]^ In recent years, research communities have witnessed a growing interest in employing plasmonic nanoparticles as photothermal agents for various applications, including photothermal catalysis,^[^ ^4^ ^]^ water purification,^[^ ^5^ ^]^ bio‐imaging,^[^ ^6^ ^]^ and most notably, for cancer photothermal therapy (PTT).^[^ ^7^ ^]^ Among various thermoplasmonic agents, gold nanoparticles (AuNPs) are particularly distinguished by their exceptional efficacy in photothermal applications and their extensive use in various biomedical fields.^[^ ^8^, ^9^ ^]^ This prominence stems from their scalable synthesis, biocompatibility, high stability, and tunable surface chemistry. The latter one enables the diverse morphologies ranging from simple spheres to complex geometries, such as nanorods and nanostars, with tunable plasmon resonances in the near infrared (NIR) region (700–1300 nm), where optical transparency in tissues makes them ideal for clinical‐related technologies.^[^ ^10^, ^11^, ^12^, ^13^, ^14^ ^]^ Unlike spherical AuNPs, whose plasmonic features are confined mainly to the visible part of the spectrum, gold nanostarts (AuNSs) have exhibited several unique advantages: (i) distinctive surface curvatures (neutral, negative, positive) which provide varied local environments for ligand attachment; (ii) branch‐dependent plasmon tunability; and (iii) intensified electric fields with a dominant number of plasmonic hotspots compared to any other synthesized plasmonic nanostructures.^[^ ^15^, ^16^ ^]^ These outstanding characteristics, combined with improvements in their synthetic approaches, render AuNSs highly promising thermoplasmonic nanoconstructs for application such as photothermal oncotherapy,^[^ ^17^ ^]^ and photoacoustic imaging.^[^ ^18^ ^]^ However, such applications require active and selective targeting of cancer cells, while sparing healthy ones. Achieving active targeting is crucial when using laser irradiation (e.g., in PPT), as it enables a reduction in the laser power, energy levels, and heat delivery required to achieve desired therapeutic effects, while ensuring adherence with medical safety standards.^[^ ^19^ ^]^ Humanized monoclonal antibodies,^[^ ^20^ ^]^ or antibody fragments,^[^ ^21^ ^]^ such as single‐chain fragment variable (ScFv), are examples of such targeting moieties particularly used in cancer research. In recent years, it has become increasingly clear that the microenvironment is essential for the tumor growth, immune suppression, invasion, and metastasis.^[^ ^22^, ^23^, ^24^ ^]^ Cancer‐associated fibroblasts (CAFs) are key components of the tumor microenvironment (TME) and play crucial roles in tumorigenesis and progression through multiple mechanisms and secretion of factors^[^ ^25^ ^]^ and overexpression of various proteins that are not present in normal counterparts.^[^ ^26^ ^]^ One of these proteins is the fibroblast activation protein (FAP), a type‐II transmembrane glycoprotein and a serine protease of the dipeptidyl peptidase family member. FAP is present on the cell membrane of ≈90% of human epithelial carcinomas.^[^ ^27^ ^]^ While its expression levels in epithelial cancer cells vary, it is consistently and abundantly expressed by CAFs, making it a highly specific marker for the tumor stroma.^[^ ^28^ ^]^ In the research community, FAP is widely accepted as one of the key biomarkers for identifying CAFs and is considered the most promising stromal targets for sloid tumor therapy.^[^ ^29^ ^]^ Therapeutic approaches targeting FAP are currently in the early stages of development, with various strategies being explored, including FAP‐targeted monoclonal antibodies or recombinant derivatives thereof,^[^ ^30^ ^]^ small molecule based FAP ligands including FAP inhibitors,^[^ ^31^ ^]^ DNA vaccine therapy,^[^ ^32^ ^]^ and chimeric antigen receptor (CAR) T‐cell therapy.^[^ ^33^ ^]^ Ongoing research on these treatments have highlighted their advantages and intrinsic limitations.^[^ ^34^ ^]^ For example, these systemic FAP‐targeted treatments have been associated with severe muscle damage, cachexia, and potentially lethal toxicities.^[^ ^34^, ^35^, ^36^ ^]^ To address the reported intrinsic challenges and side effects, especially for CAR T‐cell therapy, research community has proposed modular CAR T‐cell technologies also named as adaptor CAR T‐cell platforms, which offer enhanced safety and precision for treating both hematological and solid tumors.^[^ ^37^, ^38^, ^39^ ^]^ Such adaptor CAR platforms consist of two key components: i) CAR T‐cells and ii) the adaptor molecule also known as target modules (TMs). TMs for the UniCAR system consist of (i) an antitumor binding domain, usually configured as ScFv or immunoglobulin G (IgG) and (ii) a peptide epitope that is recognized by the extracellular ScFv domain of the UniCAR. The peptide epitope recognized by the UniCAR ScFv is derived from the monoclonal antibody 5B9 which recognizes a continuous epitope of the nuclear autoantigen La/SS‐B termed E5B9. Therefore, UniCAR TMs can act as a molecular bridge between UniCAR T‐cells and target cells. The short biological half‐life enables a unique “stop‐and‐go” control mechanism via regulation of the infusion of the respective TM. While CAR T‐cell therapies have demonstrated remarkable efficacy in the treatment of leukemias, there is still need to further improve the therapy of solid tumors. It is widely recognized that combinatorial therapies may help to overcome the limitations observed for all kind of monotherapies of tumors. Toward this goal, we aim to capitalize on the modularity of TMs, which enables not only the customization for various antigens, but also the combination with the biocompatible, self‐therapeutic properties of AuNPs.^[^ ^40^, ^41^ ^]^ Herein, our central goal is to employ theranostic carriers to which antibody‐based fibroblast activation protein FAP targeting modules (anti‐FAP TMs) can be attached. This way, unlabeled TMs could not only easily be modulated with respect to their half‐lives but also be equipped with additional functional features provided by the nanocarrier, extending the immunotherapeutic capabilities of the TMs, as well as enabling additional applications such as multimodal imaging and PPT. In this work, we demonstrate the potential of 20 nm AuNPs, both spherical and star‐shaped, as nanocarriers for anti‐FAP TMs (ScFv and IgG4).^[^ ^39^ ^]^ Employing site‐directed conjugation strategy (**Scheme** **1**a), we achieved the oriented immobilization of the targeting ligands, significantly enhancing selectivity and binding affinity. Both TM formats offer distinct advantages: The compact size of ScFvs (≈35 kDA) enables high‐density conjugation on AuNP surfaces, while the larger IgG4 (≈112 kDa) provides stronger binding affinity and prolonged systemic circulation. After thorough characterization of the nanocarriers, we assessed the optically induced photothermal heating properties of the colloids using a radiometric camera, and thermal lens spectroscopy (TLS) technique.^[^ ^42^, ^43^ ^]^ TLS proved effective in quantitatively assessing light‐to‐heat conversion efficiency, a critical parameter for optimizing nanovectors for PTT applications. Nontoxicity profile of the nanoconjugates was first assessed prior to the specificity in targeting hFAP cells, which was validated by fluorescence activated cell sorting (FACS), immunostaining, and SPRS imaging. The results demonstrate the potential of this approach as a multifunctional targeting platform that synergizes immunotherapeutics with thermoplasmonic nanocarriers, offering a promising platform for FAP‐targeted theranostics.

## 2 Results and Discussions

### 2.1 Synthesis, Characterization, and Biofunctionalization of AuNPs

**Figure 1:**
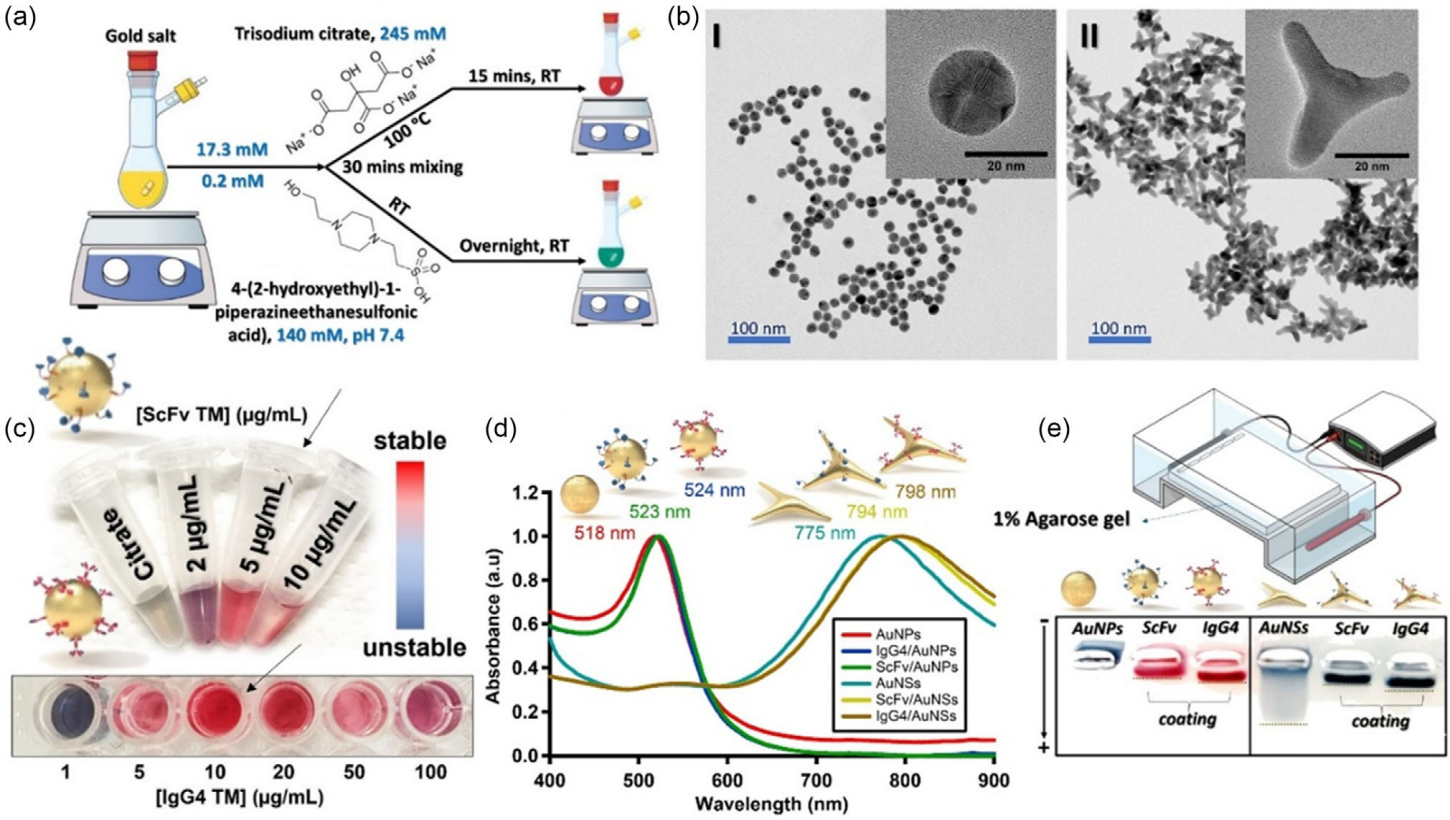
a) Schematic illustration depicts the synthetic methodologies employed in the fabrication of spherical and star‐shaped AuNPs. b) TEM micrographs displaying the morphology of the prepared particles: I) spherical AuNPs and II) AuNSs). c) digital micrographs showing the stability test for spherical AuNPs screened at varying concentrations of TM formats. The results suggest that the optimal concentrations for fully coating the AuNPs while maintaining their original red color, indicative of high stability, are 5 μg mL^−1^ for ScFv TMs and 10 μg mL^−1^ for IgG4 TMs. d) UV‐Vis absorption spectra of bare spherical AuNPs with λ_LSPR_ at 518 nm and bare AuNSs with λ_LSPR_ at 775 nm. Spherical AuNPs and AuNSs conjugated with 5 μg mL^−1^ of ScFv TMs showed redshifts of 5 nm and 19 nm in their SPR bands, respectively. When conjugated with 10 μg mL^−1^ of IgG4 TMs, the SPR bands of the spherical AuNPs and AuNSs exhibited additional redshifts of 1 nm and 5 nm, respectively. e) 1% Agarose gel electrophoresis analysis demonstrating the migration bands of coated and uncoated AuNPs. It is noticeable that the bare spherical AuNPs aggregated within the well, whereas bare AuNSs predominantly remained in the well with some smearing, indicating minimal migration and the absence of distinct bands.

The synthetic approaches for the AuNPs production are depicted in **Figure** **1**a. Spherical citrate‐stabilized AuNPs were synthesized using a modified version of the method pioneered by Turkevich et al.^[^ ^44^ ^]^ For the synthesis of AuNSs, a one‐pot seedless approach was employed utilizing 4‐2‐hydroxyethyl piperazine‐1‐ethanesulfonic acid (HEPES) and chloroauric acid (HAuCl_4_), with HEPES serving as both a reducing agent and a shape‐directing agent.^[^ ^45^ ^]^ In the context of clinical applications, where cytotoxicity is a critical concern, this nonseed‐mediated synthetic route of AuNSs using HEPES, one of the Good's buffers used in cell culture, offers a distinct advantage, providing enhanced biocompatibility and well‐defined morphological control, making them suitable for biomedical uses.^[^ ^46^, ^47^ ^]^ It is believed that the tertiary amines in the piperazine ring of HEPES generate cationic free radicals, which facilitate the reduction of gold ions, leading to the formation of AuNSs.^[^ ^48^ ^]^ Transmission electron microscopy (TEM) micrographs confirmed the spherical and branched morphologies, with diameters of ≈20 nm (Figure 1b). The average hydrodynamic diameters were measured to be 21 ± 2 nm for spherical AuNPs and 32 ± 5 nm for AuNSs, as measured by dynamic light scattering (DLS) (Figure S3, Supporting Information). The observed size variation between TEM and DLS for AuNSs can be primarily attributed to the inherent differences in measurement technique rather than actual structural variations. While TEM provides a direct measurement of the AuNSs in a dry state under vacuum, DLS measures the hydrodynamic diameter in a hydrated, colloidal state, which includes contributions from surrounding solvation layers.^[^ ^49^ ^]^ In addition, the anisotropic and multibranched morphology of AuNSs can lead to an overestimation of size in DLS due to enhanced scattering from elongated features. Both citrate‐capped AuNPs and HEPES‐capped AuNSs demonstrated excellent stability at 4 °C for one month when stored in their respective buffer growth solutions. In the case of spherical AuNPs, citrate ions play a key role in stabilization by providing electrostatic repulsion, which prevents aggregation. For AuNSs, the ethanesulfonate group of HEPES interacts with the particle surface, enhancing their structural stability. In addition, the hydroxyl group in HEPES promotes self‐assembly and bilayer formation through hydrogen bonding, further contributing to the structural stability of the AuNSs.^[^ ^45^ ^]^ It is worth mentioning that these capping agents can be easily replaced with other ligands, enabling further functionalization. The particles were then conjugated with anti‐FAP TMs through an exchange reaction that replaced the initial capping layers with Cysteine (Cys) residues at the peptide epitope, forming a self‐assembled protein monolayer (as displayed in Scheme 1b). The covalent attachment of biomolecules to Au surfaces via reactive Cys residues has been demonstrated.^[^ ^50^, ^51^ ^]^ Unbound TMs were separated from the nanoconjugates by centrifugation (14 000 rpm for 10 min). To ensure the complete removal of free TMs, multiple rounds of centrifugation and washing were performed using Tris‐HCl buffer (10 mm, pH 8.5). Proteins covalently bound to AuNPs remain attached even under conditions of protein denaturation or high salt concentration, unlike chemisorbed proteins, frequently being detached by such treatments. It is known that the introduction of high salt concentrations to AuNPs that are not fully passivated can induce their aggregation, a phenomenon that is readily observed as a color change from ruby red to blue, which is used as an indicator of the colloidal stability and presence of the proteins at the surface of the NPs. The optimal protein concentration for coupling TMs formats to spherical AuNPs and AuNSs was determined through a two‐step process. Initially, spherical AuNPs were incubated with the E5B9 peptide epitope linker. A broad concentration range of E5B9 linker (0.05 to 80 μg mL^−1^) was tested for coupling, and evaluated using ultraviolet‐visible (UV‐Vis) absorption spectroscopy. Stability testing by redispersion of the conjugates in PBS indicated that particle aggregation occurred at concentrations below 5 μg mL^−1^ (Figure S4, Supporting Information). Therefore, 5 μg/mL was identified as the optimal concentration to fully passivate the AuNPs with the E5B9 linker. Next, a narrower concentration range (2, 5, and 10 μg mL^−1^) was screened for the ScFv TM. For this TM, 5 μg mL^−1^ is the maximum concentration that can be conjugated to the nanoparticles without causing aggregation (Figure 1c). A similar optimization procedure was applied to couple the IgG4 TM to spherical AuNPs screening a broader concentration range. In comparison with the ScFv TM, higher concentrations (10 or 20 μg mL^−1^) could be coupled without aggregation. Therefore, 10 μg mL^−1^ was chosen for further experiments. The nanoconjugates, especially IgG4/AuNPs, exhibited exceptional stability maintaining their structural integrity even after a freeze‐drying cycle (Figure S5, Supporting Information), overcoming a key limitation in the widespread application of AuNPs. Figure 1d displays the UV‐Vis absorption spectra for AuNPs biofunctionalized with ScFv and IgG4, showing a localized surface plasmon resonance (LSPR) band (λ_LSPR_) redshifted from 518 nm to 523 nm and 524 nm for spherical AuNPs and from 775 nm to 794 nm and 798 nm for AuNSs, respectively. The successful coating of the TMs on the AuNPs was also confirmed by gel electrophoresis, comparing bare AuNPs as controls with AuNPs coated with ScFv and IgG4 TMs (Figure 1e). Due to their inherent colors, no dye was needed to track the mobility of the NPs along the gel. The citrate‐capped AuNPs did not migrate at all and aggregated in the running buffer (1X TAE, pH ≈ 8.0), which contained salts that led to charge screening and eventually aggregation of the particles due to attractive Van der Waals forces. In contrast, the movement of ScFv/AuNPs and IgG4/AuNPs was clearly visible to the naked eye retaining their original colors, thus indicating the presence of a coating protein layer on the nanoparticles. It is worth mentioning that the coated AuNPs exhibited less migration along the gel compared to E5B9/AuNPs (data not shown), further supporting the successful functionalization with the TMs. Sodium dodecyl‐sulfate polyacrylamide gel electrophoresis (SDS‐PAGE) (details in S6, Supporting Information) was also employed to track the mobility of the proteins conjugated to the AuNPs, based on their molecular weights. Under reducing conditions, we anticipated the cleavage of disulfide bonds within the native homodimers of the TMs, as well as with dissociation of the nanoparticle‐antibody complexes. As observed in Figure S6, Supporting Information, prominent bands corresponding to the small and full‐sized antibodies were observed at ≈35–65 kDa, respectively. Notably, the observed molecular weight of the IgG4 TM was lower than the theoretical value of 112 kDa, likely due to reduction‐induced dissociation of the homodimer into its monomeric form, yielding a band at half the expected molecular weight. In addition, AuNPs did not migrate through the gel matrix due to their large sizes and remained concentrated in the loading wells.

### 2.2 Photothermal Effect and Thermal Lens Spectrometric Setups and Measurements

**Figure 2:**
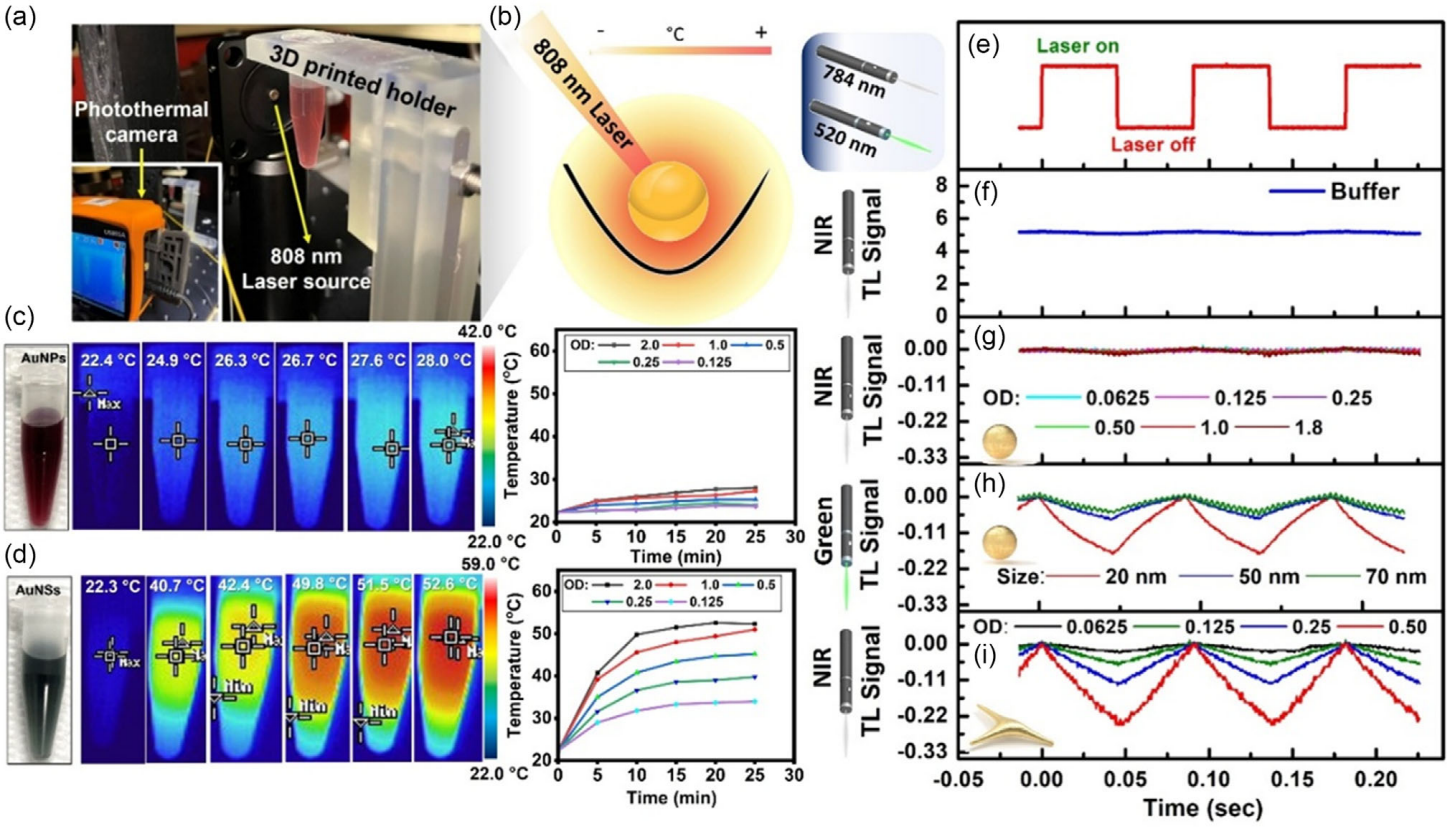
a) Digital photograph of the photothermal experimental setup showing the 808 nm laser source directed toward the AuNP sample, which is secured in place using a 3D‐printed holder, and an IR camera positioned at an optimal angle for recording the heat conversion process. b) Schematic representation of the thermoplasmonic heating of AuNPs under irradiation from a NIR laser source; notice the generation of a spatial distribution of temperature at distance from the NP surface. c,d) IR images and corresponding temperature profiles as a function of time for the AuNP and AuNSs samples (OD = 2), recorded over 25 min. e,f) TL spectroscopic profile of AuNPs at the onset of irradiation (50 ms); (e) laser on–off cycle; (f) Tris‐HCl buffer excited at 784 nm; g) spherical AuNPs at varying concentrations excited at 784 nm; and h) as a function of particle size, excited at 520 nm, i) AuNSs at different concentrations excited at 784 nm.

We further investigated the photothermal properties of the AuNPs by irradiating them with a continuous wave (CW) NIR‐I laser at 808 nm and a power density of 0.96 mW cm^2^, while recording temperature changes using an infrared (IR) thermal camera (**Figure** **2**a,b). Following 25 min of laser irradiation, no significant temperature change was detected in the control sample (Tris‐HCl buffer solution). However, the temperature of the AuNSs suspension increased to nearly 53 °C, whereas the temperature of the monodispersed spherical AuNPs reached only 28 °C (Figure 2c,d). This difference is expected, as the temperature elevation is directly influenced by LSPR and the excitation wavelength. The closer the LSPR is to the laser wavelength (as with AuNSs), the more efficient the optical‐to‐thermal energy conversion.^[^ ^52^ ^]^ Due to the mismatch between the LSPR of the spherical AuNPs (518 nm) and the laser wavelength (808 nm), compared to the AuNSs (775 nm), the AuNSs demonstrate a higher temperature increase than spherical AuNPs at the same size and concentration. While other anisotropic nanostructures such as gold nanorods (AuNRs) and gold bipyramids (AuBPs) may also exhibit enhanced optical properties in the NIR range at their respective LSPR wavelengths,^[^ ^53^, ^54^ ^]^ this study also accounts for additional factors, including bioconjugation efficiency and targeting performance. Given these considerations, spherical AuNPs were selected as a control due to their well‐characterized optical and thermal properties, surface chemistry, high stability, and widespread use in bioconjugation, offering a reliable reference system. Figure 2e–i presents the TL signal measurements at the onset of irradiation (50 ms). As shown in Figure 2f, irradiation in buffer alone did not result in any measurable temperature change. Figure 2g shows the photothermal signal of spherical AuNPs excited at 784 nm. One can notice that the TL signal of the spherical AuNPs exhibits a lower amplitude and higher noise (enlarged in Figure S7a, Supporting Information), which can be attributed to the lower absorption and photothermal conversion efficiency (PCE) of spherical AuNPs at this wavelength. Conversely, the PCE of spherical AuNPs is significantly enhanced when excited at their LSPR wavelength of 520 nm, as demonstrated in Figure 2h. To also correlate the influence of different spherical AuNPs sizes on the TL signal upon 520 nm excitation, additional spherical AuNP sizes (50–70 nm) were tested and compared to 20 nm particles. Upon laser excitation, we observed a pronounced exponential decrease in the TL signal with increasing AuNP size (Figure S7b, Supporting Information), indicating an enhancement in PCE for smaller particles. This enhanced efficiency is likely attributed to the stronger LSPR in smaller spherical AuNPs, which facilitates more efficient light absorption and heat generation, leading to superior photothermal performance at this wavelength. Interestingly, as shown in Figure 2i, even at a lower concentration (OD = 0.5), AuNSs exhibit higher PCE (TL signal = −0.24) when excited at 784 nm compared to similarly sized spherical AuNPs at a higher concentration (OD = 1.0) with a TL signal of −0.16 under excitation at 520 nm (Figure 2h). These findings highlight the superior photothermal conversion capabilities of AuNSs, demonstrating a 1.5‐fold enhancement compared to spherical AuNPs, even at the very beginning of the irradiation period (50 ms). For clinical applications, the selection of an optimal thermoplasmonic agent and the corresponding laser wavelength for PTT application is critically dependent on the cancer type and its anatomical location. For instance, in the treatment of superficial malignancies, where visible CW lasers are commonly used, spherical AuNPs have demonstrated effective photothermal performance, as previously reported by earlier work of Elsayed and co‐workers.^[^ ^55^ ^]^ In contrast, for deep‐seated tumors, AuNSs offer a superior alternative due to their strong absorption in NIR region, which allows for greater tissue penetration compared to spherical AuNPs. As shown in Figure 2, AuNSs exhibit significantly higher PCE than spherical AuNPs of similar size. Along this line, direct PCE measurements have been reported for 30 nm and 60 nm AuNSs, with comparisons to gold nanoshells, one of the most well‐studied nanostructures used for PTT.^[^ ^56^ ^]^ At equivalent ODs, 30 nm AuNSs exhibited the highest efficiency in converting the incident laser power into heat, consistent with the theoretical predictions, indicating that AuNSs possess a substantially higher absorption‐to‐scattering ratio compared to other gold nanostructures, including gold nanoshells.^[^ ^57^ ^]^ Such high conversion efficiency is particularly advantageous for in vivo applications. In the same study, the in vivo PTT results have demonstrated that a single 20‐min laser treatment (0.7 W cm^2^) can effectively ablate highly aggressive primary soft‐tissue sarcoma loaded with AuNSs with higher tumor uptake of 30 nm AuNSs than that of 60 nm AuNSs with the same injection dose of gold (200 μg).^[^ ^56^ ^]^ It is important to mention that the 980 nm laser used in this study was selected to match the 945 nm plasmon peak of the 30 nm AunNSs; however, this wavelength also overlaps with the absorption band of water, which likely contributed to the mild skin burns observed in mice. To address this limitation, it has been recommended to tune the LSPR peak of AuNSs to lower wavelengths (≈800 nm), where water absorption is significantly reduced. The LSPR of the AuNSs‐based nanoconjugates presented in this study falls within this optimal spectral range, highlighting their potential for future in vivo PTT applications.

### 2.3 FACS, Immunostaining, and SPR Analysis for Specific Cell‐Nanoparticle Interactions

**Figure 3:**
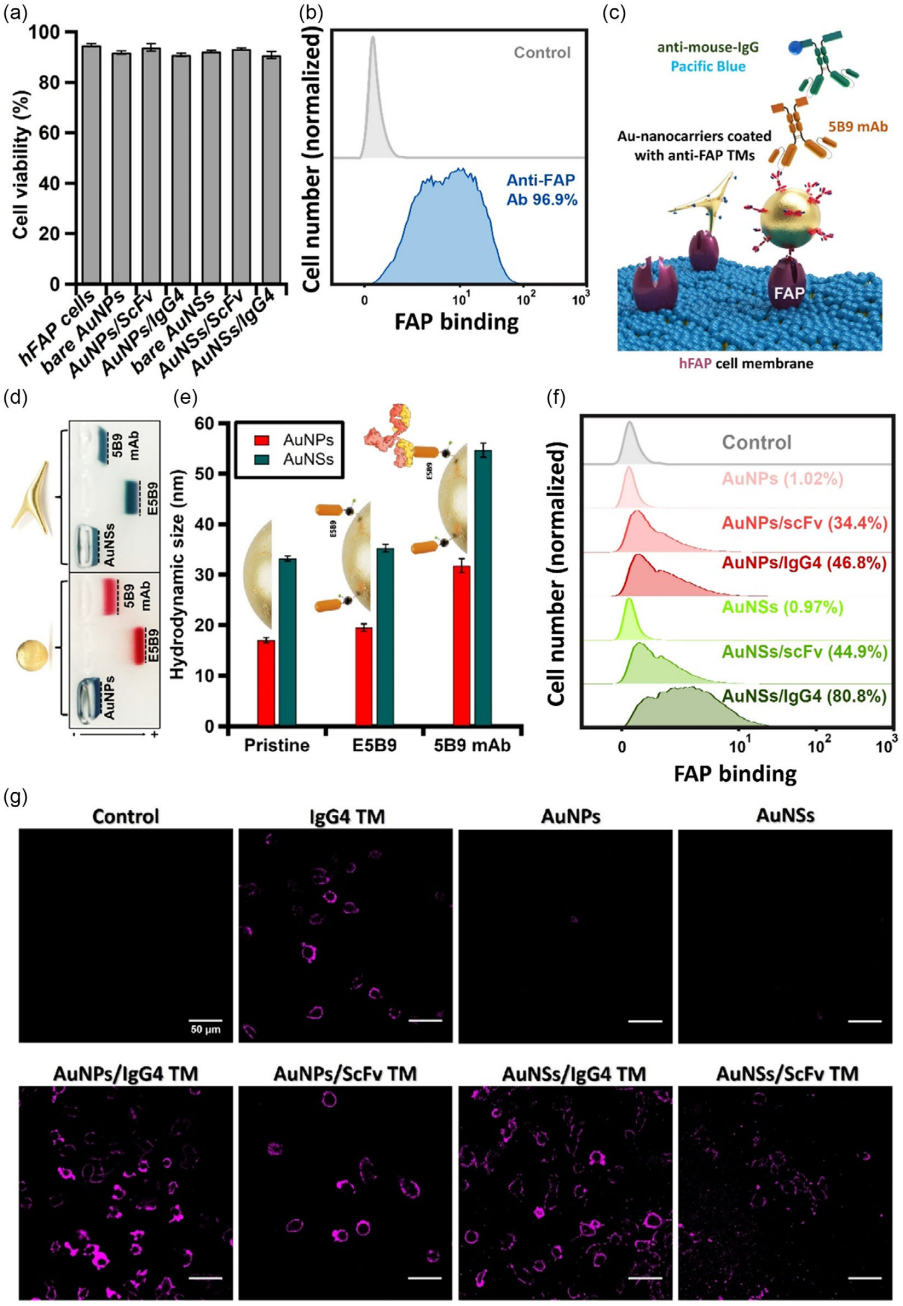
a) Viability assessment of the cells following treatment with nanoconjugates (OD = 0.1). b) Surface expression of hFAP on HT1080 hFAP cells assessed by FACS using the commercial anti‐FAP 5b9 mAb (*n *= 1). c) Schematic illustration of the fluorescent‐based assay used for the FACS experiment. d) Gel electrophoresis analysis demonstrating the migration bands of bare AuNPs, E5B9, and 5B9‐coated AuNPs. e) Volume‐weighted DLS readouts showing the size evolution of AuNPs and AuNSs coated with E5B9 and 5B9. f) FAP‐specific binding of the nanoconjugates to hFAP cells evaluated by FACSs (*n* = 1). g) Fluorescence microscopy images of hFAP cells at different conditions. Scale bars: 50 μm.

To understand if the AuNPs and their surface modifications with the TMs can affect cell proliferation and death, cell viability using hFAP cells was analyzed with a Casy‐ton cell counter. The results in **Figure** **3**a show that the number of viable cells was always >90% for hFAP cells in both the control and treated groups after 24 incubations with nanoconjugates, indicating no sign of cytotoxicity. This observation is consistent with previous reports on AuNPs, as similar‐sized nanospheres (15 nm) have been approved by the FDA for use as contrast agents in diagnostic imaging.^[^ ^58^ ^]^ Similarly, our findings for AuNSs align also with existing literature demonstrating their low cytotoxicity compared to other anisotropic nanostructures.^[^ ^59^, ^60^ ^]^ It is also well known that cytotoxicity of AuNPs is both dose‐ and exposure time‐dependent. In this study, the used nanoconjugates concentration (0.1 OD ≈ 0.4 nM) falls within the range of biologically safe doses, further supporting the observed low cytotoxicity.^[^ ^61^ ^]^ To further evaluate the FAP protein targeting capabilities of the nanoconjugates, their specific interaction with hFAP cells was investigated. While free anti‐FAP TMs exhibit a high affinity for the FAP receptor,^[^ ^39^ ^]^ it was essential to confirm whether its binding is retained following conjugation to the nanoparticles. The overexpression of hFAP on the surface of the modified HT1080 hFAP cells was confirmed using FACS (Figure 3b). Then, the binding of the nanoconjugates to hFAP cells was also analyzed. As depicted in Figure 3c, the nanoconjugates targeting the FAP receptor were probed with a primary anti‐E5B9 monoclonal mouse IgG (5B9), which specifically recognizes the E5B9 epitope of the TMs. The detection was achieved using a secondary goat anti‐mouse IgG antibody conjugated to the fluorescent dye Pacific Blue emitting at 455 nm (Invitrogen, #P31582), and the resulting fluorescence signal was quantified by FACS analysis. A key concern prior this assay was the potential steric hindrance of the E5B9 peptide epitope caused by the attachment of TMs to AuNPs, which could hinder the antibody recognition. To evaluate this, the accessibility of the E5B9 epitope on the nanoconjugates was evaluated through gel electrophoresis and DLS. As shown in Figure 3d, 5B9‐decorated AuNPs band showed a reduced electrophoretic mobility relative to E5B9/AuNPs band due to the alteration in the size and charge of the E5B9‐ attached AuNPs, indicating successful antibody binding. This was further supported by DLS readouts (Figure 3e), showing a sequential increase in average particle size, indicating the functionality of the E5B9 on the nanoconjugates. Following this validation, hFAP cells were incubated with different sample types: bare nanocarriers (negative controls) and nanoconjugates with either TM on each nanocarrier. The FACS results presented in Figure 3f demonstrated a significant shift in fluorescence intensity when hFAP cells were incubated with the nanoconjugates, indicating that the TMs retained high and specific binding to hFAP cells after conjugation. Notably, the nanoconjugates based‐AuNSs exhibited higher binding efficiency compared to those based on spherical AuNPs for both TMs. This observation is consistent with findings previously reported.^[^ ^62^ ^]^ This enhancement is attributed to the architecture of the AuNSs, which provides multiple points of contact and enhanced ligand presentation, enabling multivalent interactions between the targeting vectors and its receptor, whereas spherical AuNPs offer only a limited area for ligand‐receptor contact. Furthermore, nanoconjugates incorporating IgG4 TMs exhibited superior binding compared to those based on ScFv TM. This finding is expected, considering the structural advantages of IgG4 TM which possess bivalency, and greater conformational stability relative to the smaller ScFv TM. In addition, the IgG4 TMs are engineered with one E5B9 epitope per heavy chain,^[^ ^63^ ^]^ a design feature that likely enhances the anchoring efficiency of IgG4 TMs on the nanocarrier surface and facilitates more effective receptor engagement. To further validate the FACS findings, immunofluorescence microscopy was performed on samples prepared similarly to those used for FACS analysis, with the exception that the secondary antibody was replaced by a chicken anti‐mouse IgG conjugated to a 647 nm‐emitting dye. As anticipated, no detectable fluorescence signal was observed in any of the samples in the absence of the 5B9 primary antibody (Figure S9, Supporting Information). In contrast, as shown in Figure 3g, a significant fluorescence signal was detected in fully stained samples when hFAP cells were incubated with the free TMs, the nanoconjugates, but not with the bare AuNPs, further corroborating the findings observed by FACS. Overall, these results not only confirm the specific binding capabilities of the nanoconjugates to FAP‐expressing cells, but also emphasize the critical role of nanocarrier morphology and targeting moiety architecture in cellular targeting efficiency.

**Figure 4:**
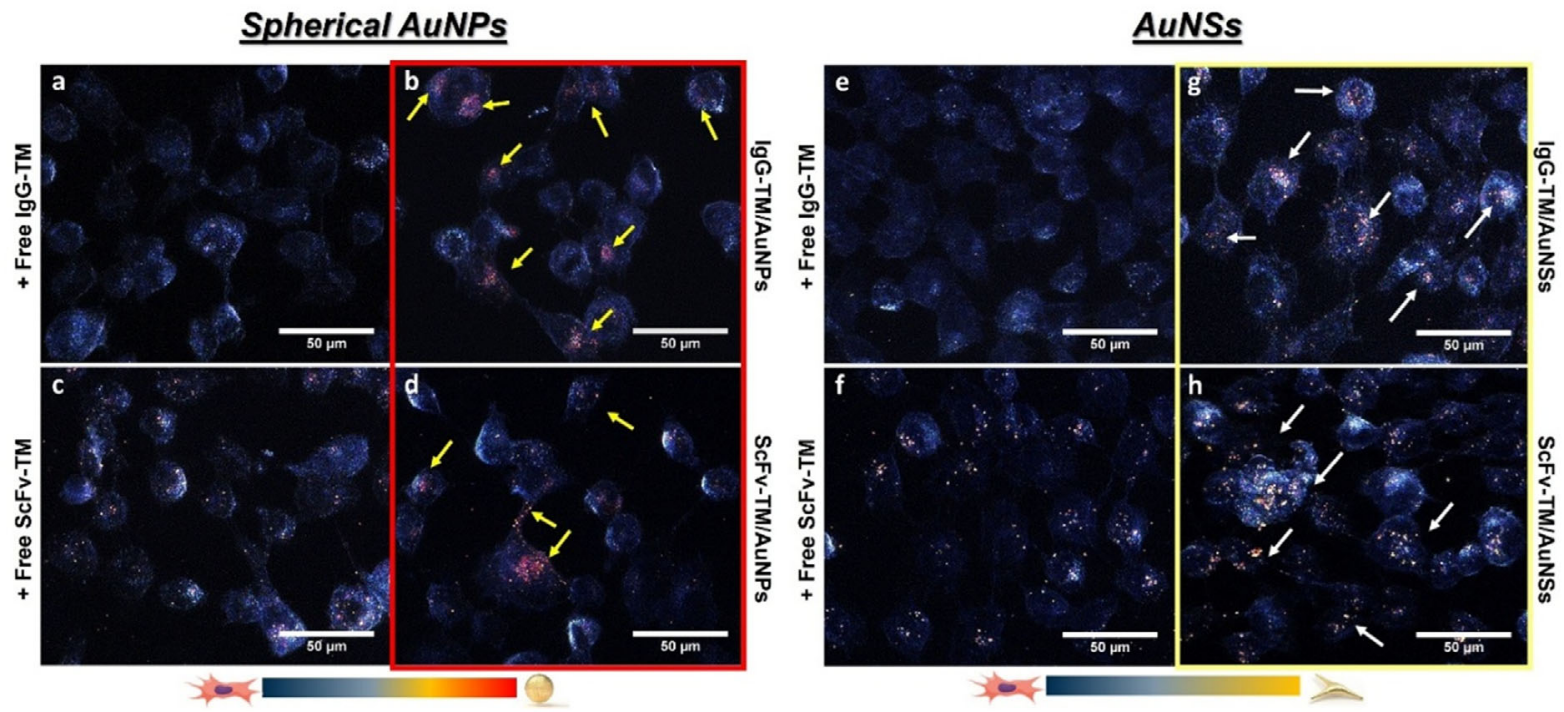
Light‐scattered images of hFAP cells following two hours of incubation with OD 0.75 of: a) AuNPs/IgG4 with an excess IgG4, b) AuNPs/ScFv with an excess of ScFv, c) AuNP/IgG4, d) AuNP/ScFv, e) AuNSs/IgG4 with an excess IgG4, f) AuNS/ScFv with an excess of ScFv, g) AuNS/IgG4, and h) AuNS/ScFv. The color scale at the bottom indicates the spectral scattering profiles: Blue represents cellular structures and organelles. Reddish hues correspond to the scattering of spherical AuNPs. Yellowish hues indicate the scattering of AuNSs. Yellow and white arrows highlight regions of increased nanoparticle accumulation. Scale bars: 50 μm.

To explore the specific interactions between cells and nanoparticles, we devised an experiment using the unique light absorption and scattering properties of AuNPs. AuNPs are known to scatter light of various colors when illuminated with white light at specific angles. Such size‐ and shape‐dependent scattering offers the potential for labeling applications in cellular imaging and detection using a white light source. Unlike our previous experiment, where cells were fixed before treatment, in this study, the AuNPs were introduced into living cells via endocytosis during the processes of cell differentiation and proliferation. SPRS images were then recorded after treatment using the partial ring illumination mode in the digital microscope (Keyence VHX‐X1, Japan). In this mode, only scattered light is collected, resulting in an image of bright objects against a dark background. **Figure** **4** displays the light scattering images of hFAP cells incubated with four different treatments (Figure S10, Supporting Information). All cells displayed white and blue scattered light, which can be attributed to autofluorescence and scattering from cellular organelles. The spherical AuNPs exhibit a distinct reddish color due to their strong LSPR absorption in the visible spectrum. On the other hand, AuNSs appear yellowish‐orange, a result of their branched morphology, which shifts their LSPR toward the NIR window, instead of the typical red color associated with smaller spherical AuNPs. When hFAP cells were incubated with TM‐coated AuNPs and 1 μg/mL of the corresponding free TMs, almost no particle signal was detected in cells (Figure 4a,e).

In contrast, some particles were observed in cells treated with AuNPs/ScFv, even in the presence of excess ScFv (Figure 4c,f). This could be attributed to the higher density of ScFv molecules attached to the AuNPs, due to their smaller size. These findings suggest that, in the case of free IgG4, all antigen‐binding sites on the cells were saturated, whereas in the case of ScFv, competition with the free protein may have reduced the accessibility of the antigen recognition sites on the ScFv‐coated AuNPs. In the case of direct incubation of hFAP cells with TM‐coated AuNPs (Figure 4b,d,g,h), a higher density of nanoparticles was clearly observed within the cells, evidenced by the increased red and yellow scattered light from spherical AuNPs and AuNSs. Interactions with PC3 cells, employed as a negative control, as no FAP is expressed in this cell line (Figure S11a, Supporting Information), revealed no discernible increase in red/yellow scattering intensity (Figure S11b, Supporting Information), indicating the capability of the nanoconjugates for specific cell targeting.

## 3 Conclusion

Designing intelligent tumor targeting AuNP‐assisted PTT either alone or in combination with other therapies, such as immunotherapy, holds promise for combating cancer and metastasis. Among Au‐based thermoplasmonic agents, AuNSs have gained particular attention for their biosafety, facile synthesis, and tunable photothermal properties. In this study, we developed and compared FAP‐targeting thermoplasmonic nanoconjugates by incorporating novel immunotherapeutic TMs designed against FAP and Au‐based nanocarriers for combinatorial PTT and immunotherapy. We developed AuNSs with strong absorption for the 808 nm NIR laser and superior PCE compared to similarly sized spherical AuNPs. The prepared nanoconjugates demonstrated excellent colloidal stability, protein immobilization capacity, and selective binding to FAP‐overexpressing cancer cells with minimal off‐target interactions observed in control groups. Our results suggest that the combination of thermoplasmonic agents with anti‐FAP TMs with proven specific accumulation at FAP‐expressing sites pave the way to a new era of cancer theranostics for solid tumors. Ongoing experiments are exploring the radiolabeled anti‐FAP TMs for coating the nanocarriers for multimodal imaging applications. Future investigations are warranted to explore the optimal protocols for in vivo applications, including toxicity, biodistribution, and pharmacokinetics to optimize clinical translation.

## 4 Experimental Section

4.1

4.1.1

##### Synthesis and Characterization of AuNPs and AuNSs

20 nm spherical AuNPs were synthesized via sodium citrate reduction of hydrogen tetrachloroaurate (HAuCl_4_, Sigma‐Aldrich) following a previously published protocol.^[^ ^64^ ^]^ Briefly, 1.1 mL of 17.3 mm HAuCl_4_ was added to 43.6 mL of boiling Milli‐Q under stirring. Then, 300 μL of 0.245 m trisodium citrate dihydrate (Sigma–Aldrich) was rapidly added under vigorous stirring. The reaction mixture underwent a color transition from yellow to colorless, then dark blue, and finally deep ruby red, confirming the formation of spherical AuNPs. The reaction was maintained at boiling for additional 30 min to assure the complete reduction of the gold salt, followed by at room temperature (RT). AuNPs with diameters of 50 nm and 70 nm were purchased from Sigma–Aldrich and Cytodiagnostics (Burlington, ON, Canada), respectively. The AuNSs were synthesized using a previously reported seedless method.^[^ ^45^ ^]^ A 25 mL of 140 mM (pH 7.2) (HEPES buffer, Sigma–Aldrich) was stirred for 5 min, followed by the addition of 289 μL (17.3 mM) (HAuCl_4_, Sigma–Aldrich). The molar ratio of precursors was maintained at 700:1. The mixture was stirred for 30 min and then left undisturbed at RT overnight. The optical profiles, the morphologies, and the hydrodynamic diameters of the AuNPs were verified by UV‐Vis spectroscopy, TEM (image‐Cs‐corrected Titan 80‐300, FEI Company, Eindhoven, the Netherlands), and dynamic light scattering (DLS, Malvern Instrument, UK), respectively.

##### Functionalization of AuNPs and AuNSs with anti‐FAP TMs

To functionalize the AuNPs with TMs, AuNPs were centrifuged at 14 000 rpm for 10 min. The supernatant was discarded, and the particles were resuspended in 200 μL of Tris‐HCl buffer (10 mM, pH 8.5) containing 5 μg mL^−1^ of ScFv or 10 μg mL^−1^ of IgG4 TMs. The AuNPs concentration was standardized to an optical density (OD) of 1, corresponding to ≈1.64 × 10^12^ NPs mL^−1^. The mixtures were incubated while shaking for 1 h. After that, the nanoconjugates were centrifuged twice at 14 000 rpm for 10 min each. The supernatants were removed, and the pellets were resuspended in the desired volume of Tris‐HCl buffer (10 mm, pH 8.5) or a cell culture media. Surface functionalization was confirmed by monitoring the characteristic SPR band before and after the protein coating using UV‐Vis spectroscopy, the gel electrophoretic mobilities, and the change in the hydrodynamic diameters of the AuNPs. The resulting conjugates were stored in the same buffer at 4 °C until further use.

##### Photothermal Effect and TLS: Setups and Measurements

To characterize the photothermal effect of the AuNPs, 1 mL of spherical AuNPs or AuNSs at varying optical densities (OD 2, 1, 0.5, 0.25, and 0.125) were placed in 1.5 mL tubes and positioned in a custom‐designed 3D‐printed sample holder. The AuNPs were irradiated with an 808 nm NIR‐I laser (L808P500MM, Thorlabs). The temperature of the AuNPs solutions was monitored using an IR camera at 5 min intervals for a total duration of 25 min. As a control, Milli‐Q water was also subjected to the same experimental conditions. To complement the macroscopic observations from IR images, we employed a highly sensitive technique, referred to as TLS,^[^ ^42^ ^]^ to evaluate the microscopic thermal responses of the colloidal particles under laser irradiation with a low optical power and short illumination time. The principle of TLS is illustrated and depicted in Figure S1, Supporting Information, with the experimental setup in Figure S2, Supporting Information.

##### Cell Culture, Viability Assay, and Cell Labeling

The HT1080 fibrosarcoma cells, obtained from the American Type Culture Collection (ATCC), were transfected to overexpress human FAP (hFAP) as previously described.^[^ ^39^ ^]^ FAP expression was confirmed by flow cytometry. hFAP cells were cultured in DMEM STINO medium, consisting of Dulbecco's Modified Eagle Medium (DMEM, 4.5 g L^−1^ D‐Glucose, without Sodium Pyruvate, Gibco) supplemented with 10% fetal bovine serum (Biochrom), 1% streptomycin and penicillin (Biochrom), and 1% MEM non‐essential amino acid solution (Sigma–Aldrich) at 37 °C with 5% CO_2_, and the medium was changed every 2–3 days. Cell viability was assessed using Casy‐ton cell counter (3 × 3 cycles, measuring capillary: 150 μm) after detaching with 2 mM EDTA PBS solution for 5 min. For flow cytometric experiment, AuNPs conjugated with anti‐FAP TMs (OD 4.5) were prepared as described above. The hFAP cells were detached with PBS + 2 mM EDTA, manually counted and plated (150 000 cells per well) in a 96‐well plate (V shape). The cells were then resuspended in PBS + 2% FBS (up to 150 μL). The plate was centrifuged for at 360 × g for 3 min, 4 °C, and the supernatant was discarded by flipping the plate. Next, the hFAP cells were resuspended and incubated with 50 μL of the nanoconjugates or the bare AuNPs for 1 h at 4 °C. Following the incubation, the cells were washed with 100 μL PBS + 2% FBS, centrifuged as before, and the supernatant was discarded. Next, the cells were resuspended in 50 μL of 5B9 monoclonal antibody solution (5 μg mL^−1^ in PBS) and incubated for 30 min at 4 °C. After another wash and centrifugation cycle, the cells were resuspended in 50 μL of goat anti‐mouse IgG secondary antibody conjugated to Pacific Blue (1:1000 dilution in PBS; Miltenyi Biotec) and incubated for 30 min at 4 °C. Following three final washing steps and centrifugation, the cells are resuspended in PBS and transferred to U‐shape 96‐well plate for FACS measurements. For the immunostaining, hFAP cells, fixed with 4% paraformaldehyde in 8‐well plates, were incubated overnight at 4 °C with 200 μL of the test samples. After washing with PBS (150 mm, pH 7.4) containing 2% BSA, the cells were incubated with 5 μg mL^−1^ of the 5B9 monoclonal mouse IgG primary antibody for 1 h at RT, followed by three washes with the same buffer. Next, the cells were incubated with a 1:1000 dilution of chicken anti‐mouse IgG conjugated to a 647 nm fluorophore for 1 h at RT. After washings, samples were analyzed using fluorescence microscopy.

##### Specific Nanoparticle‐Cell Interaction Analysis via SPRS Imaging

For the SPR scattering analysis, the nanoconjugates were diluted in cell culture media to obtain a final OD of 0.75, resulting in 1 mL of diluted solution. The media in each well of 8‐well culture plates was replaced with 200 = μL of the prepared samples, and cells were incubated for 2 h in a standard incubator. Following that, the cell monolayer was rinsed three times with PBS, and fixed using 4% paraformaldehyde. The cells were then maintained in PBS for imaging. As a negative control, human prostate cancer cells (PC3) were cultured and prepared following the same protocol. Light scattering images were captured using a digital microscope (VHX‐X1, Keyence), which offers advanced imaging capabilities and high‐resolution image acquisition. The partial ring mode was used to achieve selective illumination from specific angles via a ring‐shaped light source. In this mode, only a portion of the ring light is activated, providing directional lighting that enhances the visibility of surface features.

## Conflict of Interest

The authors declare no conflict of interest.

## Supporting information

Supplementary Material

## Data Availability

The data that support the findings of this study are available from the corresponding author upon reasonable request.
