# Supplementary material for: Exploring Morphology of Thermoplasmonic Nanoparticles to Synergize Immunotherapeutic Fibroblast Activation Protein‐Positive Cell Sensitization and Photothermal Therapy
Source: Small Sci. 2025 May 26;5(8):2500099. doi: 10.1002/smsc.202500099 (PMC12362733; doi:10.1002/smsc.202500099)
Supplement: Supplementary file 1 — Supplementary Material [file SMSC-5-2500099-s001.pdf]

Synergizing Immunotherapeutic Target Modules and Thermoplasmonic Gold Nanoparticles for FAP-Positive Cells Sensitization and Photothermal Therapy

Ahmed Alsadig,[1] Xuan Peng,[1] Hugo Boutier, [1] Liliana R. Loureiro,[1] Anja Feldmann,[1] René Hübner,[2] Humberto Cabrera,[3,4] Manja Kubeil, [1,5] Michael Bachmann,[1] Larysa Baraban[1,6]**

[1]Helmholtz-Zentrum Dresden-Rossendorf, Institute of Radiopharmaceutical Cancer Research, 01328 Dresden, Germany.
[2]Institute of Ion Beam Physics and Materials Research, Helmholtz-Zenztrum Dresden-Rossendorf e. V., 01328 Dresden, Germany.
[3]MLab, STI Unit, The Abdus Salam International Centre for Theoretical Physics, 34151, Trieste, Italy
[4]PDMFC, Rua Fradesso da Silveira, n. 4, Piso 1B, 1300-609, Lisboa, Portugal
[5]Institute of Resource Ecology, Helmholtz-Zentrum Dresden-Ressensorf, 01328 Dresden, Germany
[6]Else Kröner Fresenius Center for Digital Health, Faculty of Medicine Carl Gustav Carus, Technische Universität Dresden, Dresden, Germany
*Corresponding authors:
a.alsadig@hzdr.de
l.baraban@hzdr.de

Supporting information

S1. Thermal lens spectroscopy (TLS)

As shown in Figure **S1**, TLS is based on the principle that when a sample is irradiated by a laser with a Gaussian intensity distribution, the absorbed light energy is converted into heat, creating a temperature gradient within the sample. This temperature gradient induces a corresponding gradient in the refractive index of the medium, forming a transient optical element, referred to as a thermal lens (TL). The probe beam (PB) passing through this TL experiences wavefront distortions, and the magnitude of these distortions is quantified by measuring the transmittance through a small aperture located in the far field along the PB axis. The intensity of the TL signal is directly related to the amount of heat generated in the irradiated sample, which is dependent on the

concentration of the absorbing species, as well as the light to heat conversion efficiency. Our TLS system employs a 784 nm or 520 nm diode-pumped solid-state laser (DPSS) (UltraLasers) with a maximum output power of 100 mW as an excitation source. A signal generator (SG) (Rigol DG 2041A, Batronix) modulates the excitation laser (EL) at 11 Hz, and a neutral density filter (NDF) (NDC 50S-3, Thorlabs) fix the laser power to 24.8 mW. The excitation beam is collimated using the lenses L3 (LB1027-A, f = 40 mm, Thorlabs) and L4 (LB1676-A, f = 100 mm, Thorlabs). Then, the beam is focused onto the sample using L5 (LB1811-A-ML, f = 35 mm, Thorlabs). The beam radius of the excitation beam (10 µm) was measured using a commercial dual scanning slit beam profiler (BP209-VISM, Thorlabs). The probe laser, a He-Ne laser (05-UR-111, 2 mW, Melles Griot) was collimated by lenses L3 and L4 and directed to the sample via mirror M1. The transmitted beam was then directed through a 0.5 mm pinhole and an interference filter (632.8 nm, Melles Griot) to a silicon photodetector (PDA36A-EC, Thorlabs). The analog signal from the photodiode was measured using an oscilloscope (RIGOL DS1102E). The signal $S(t)$ has a negative sign as it is defined as the relative change of the light transmission $T(t)$ through a small aperture when the excitation beam illuminates the sample, as follows[40]:

$$S(t) = \frac{T(t) - T(0)}{T(0)}$$

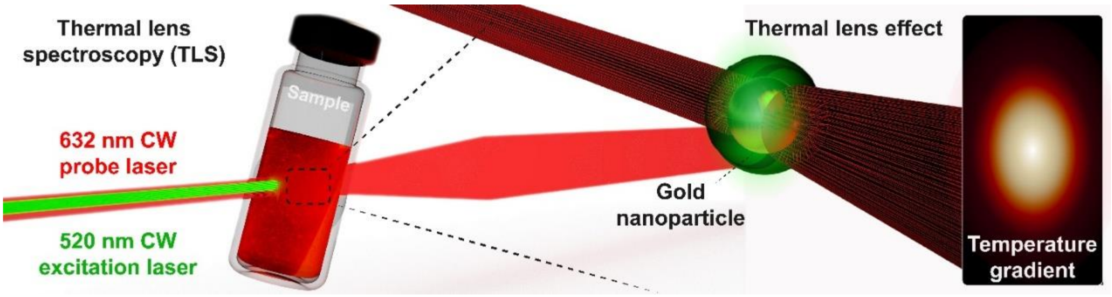

S1. Thermal lens effect; using a 632 nm laser beam to probe the relative change in the refractive index (thermal lens) of the suspension induced by a 520 nm laser beam excitation.

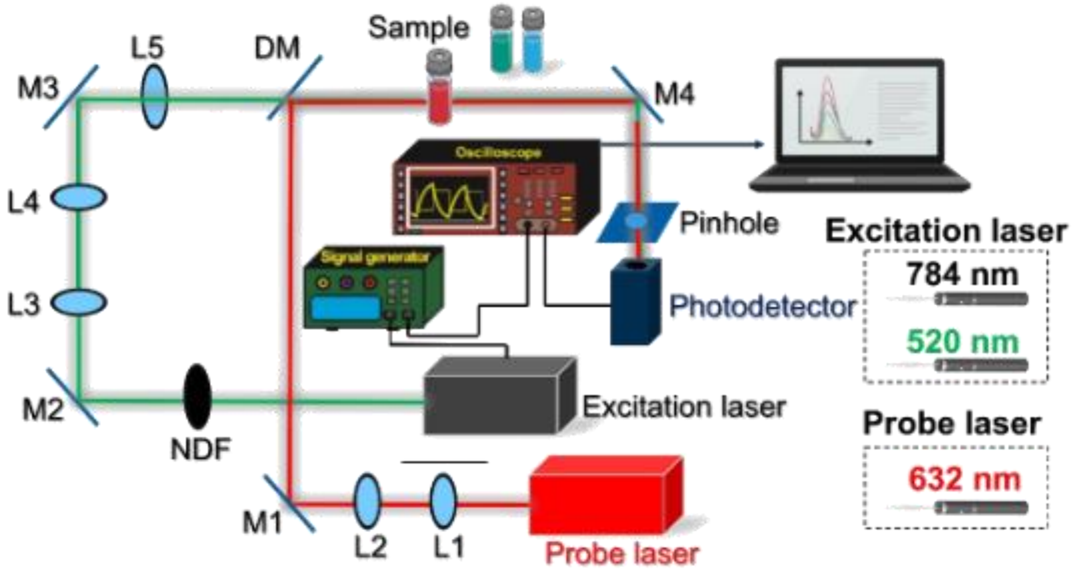

S2. TLS system experimental setup. The abbreviations denote NDF: neutral density filter, L1 to L4: lenses, M1 to M4: mirrors, DM: dichroic mirror. TL signal measured for spherical AuNPs and AuNSs excited at two distinct wavelengths.

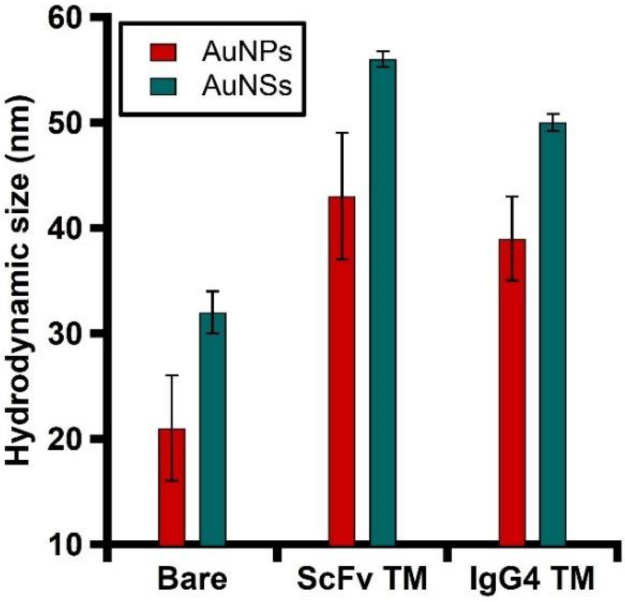

S3. Volume-weighted size distribution DLS measurements of AuNPs and AuNSs after different steps of surface coating.

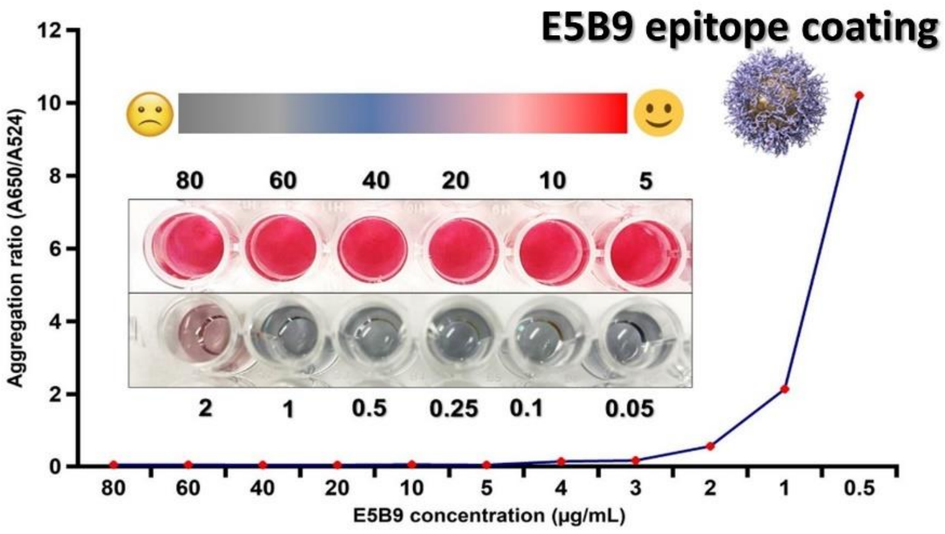

S4. Normalized aggregation ratio of AuNPs passivated with different concentrations (0.05-80 µg/mL) of E5B9 linker. The blue color indicates that the linker was insufficient to cover the surfaces of the particles. Once a sufficient amount was used, the colloidal solution does not form agglomerates, and the red color is maintained.

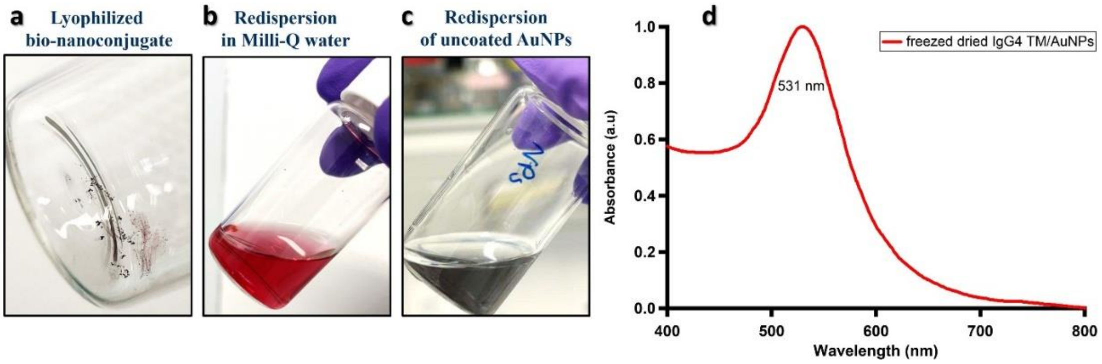

S5. Evaluation of the durability of the bio-nanoconjugate to a freeze-drying cycle. Their characteristic color change was used as an indicator. (a) Lyophilized IgG4/AuNP nanoconjugate, (b) redispersion of the bio-nanoconjugate in Milli-Q water, and (c) dispersion of unmodified AuNPs after the freeze-drying cycle, (d) Optical profile of the redispersed bio-nanoconjugate after the freeze-drying cycle.

S6. SDS-PAGE Analysis under Reducing Conditions

SDS-PAGE under reducing conditions (50 mM DTT and 5% β-mercaptoethanol) was performed. In brief, 25 µL of TMs (0.4 µg) and nanoconjugates (containing 5 and 10 µg/mL of TMs conjugated to AuNPs) were prepared and combined with 5 µL of 5X

loading buffer (125 mM Tris-HCl, pH 6.8, 40% glycerol, 0.05% (w/v) bromophenol blue, 10% SDS). The mixture was heated at 95°C for 10 minutes with occasional vortexing. Subsequently, 30 µL of each heated sample, along with molecular weight markers, was loaded onto a 10% SDS-PAGE resolving gel with a 4% stacking gel. Electrophoresis was conducted in PAGE running buffer (50 mM Tris base, 192 mM glycine, 0.1% SDS) at 120 V for 60 minutes. Following electrophoresis, the gel was stained with Coomassie Brilliant Blue G-250 to visualize protein bands

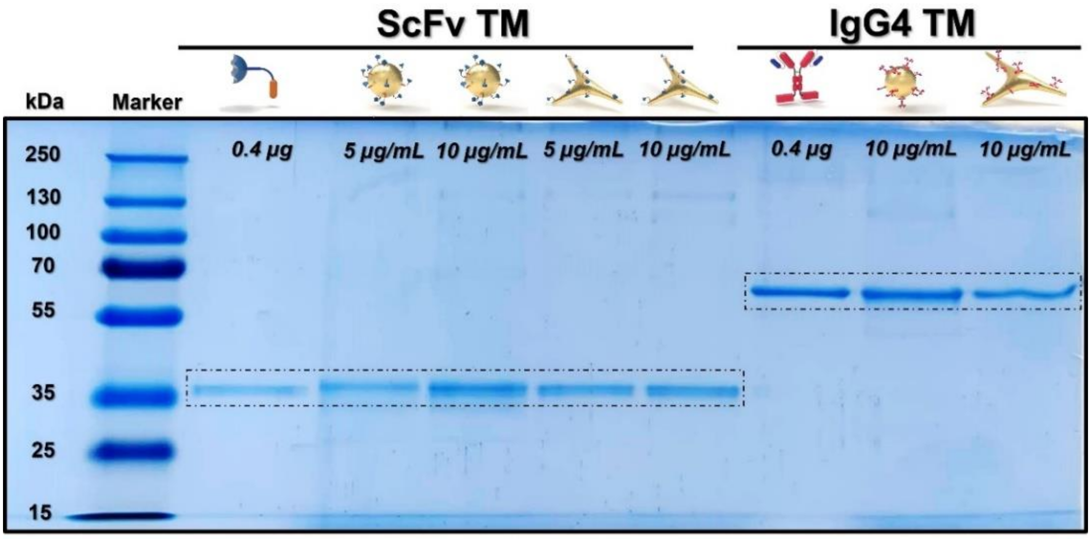

S6. SDS-PAGE analysis of the TMs and the nanoconjugates prepared under reducing conditions, followed by Coomassie Blue Staining.

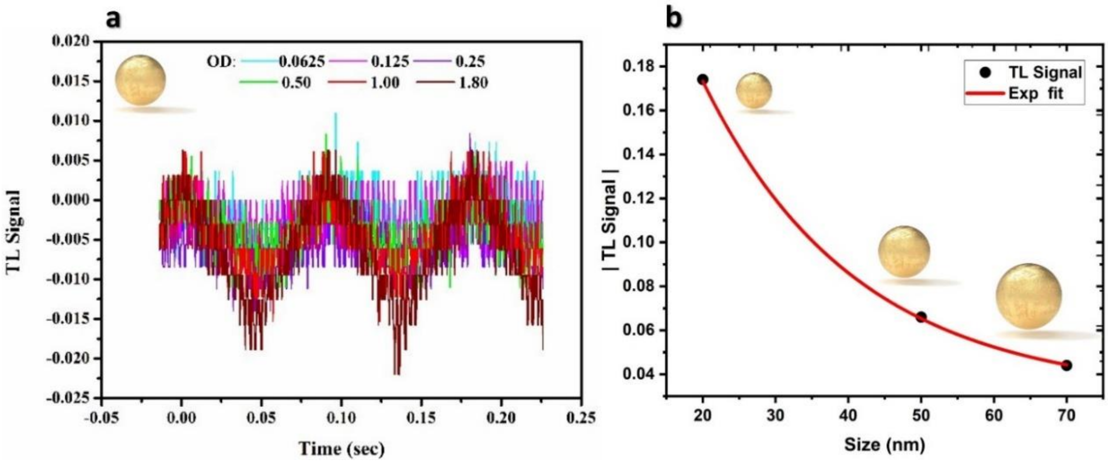

S7. (a) Magnified view of the data shown in the Figure 3g of the main text (b) Exponential plot of the modulus of the TL signal versus the particle size increase.

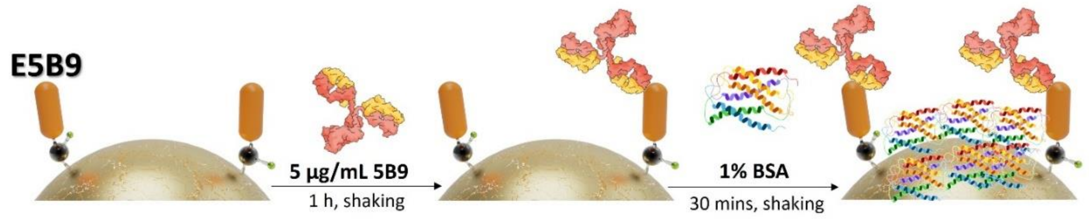

S8. The functionalization step for peptide/antibody biorecognition assay.

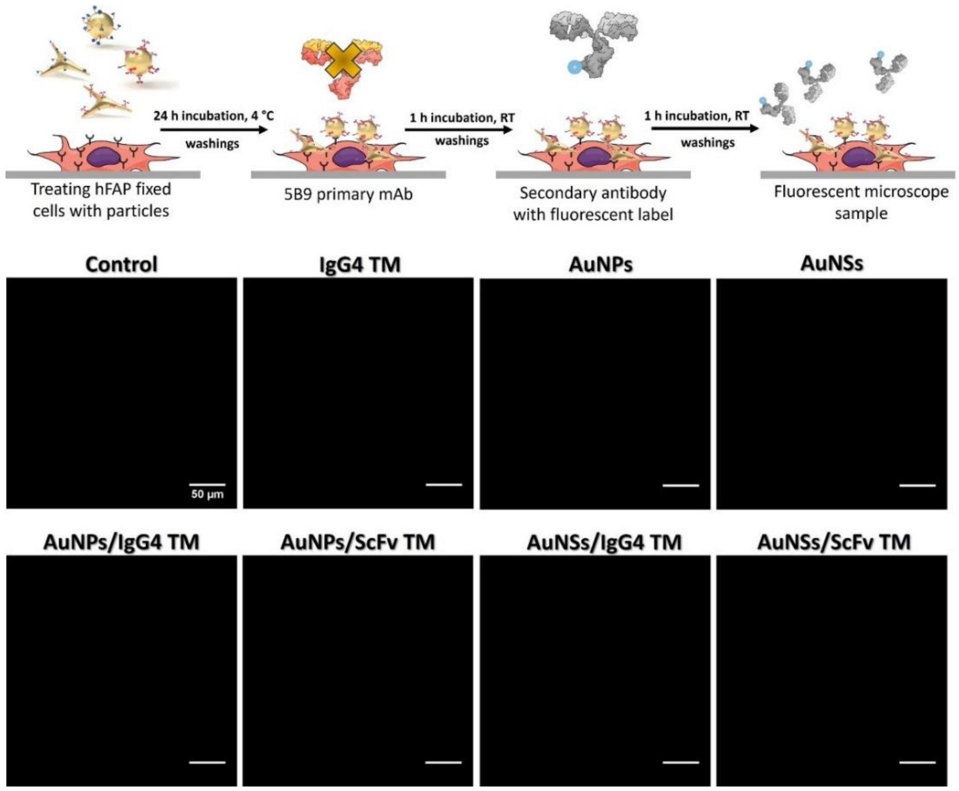

S9. Fluorescence microscopy images of hFAP cells under various conditions. In the absence of the primary antibody, the secondary antibody showed no binding to cell surface molecules, resulting in no detectable fluorescence signal across all tested samples.

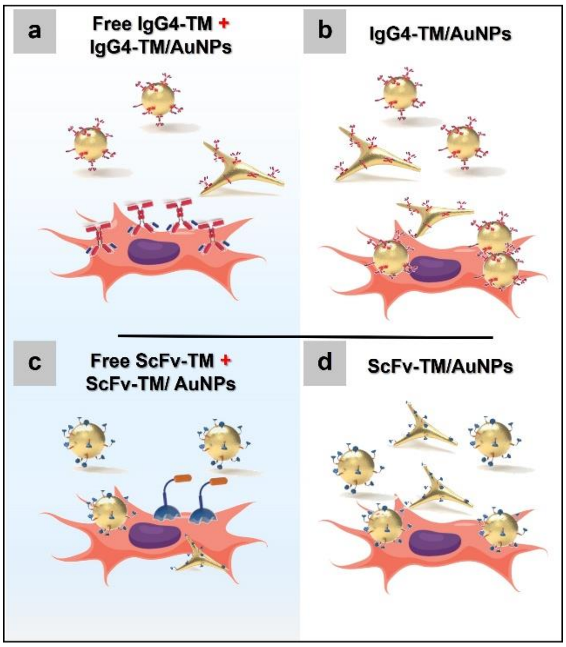

S10. Schematic representation of the experimental design to study the interaction of TMs-AuNPs and hFAP cells. In the competitive assay (a and c), cells were pre-treated for 30 mins with an excess of TMs (1 µg/mL) in two formats, IgG4 and ScFv, before the addition of AuNPs. In the non-competitive assay (b and d), cells were directly treated with AuNPs without prior exposure to TMs.

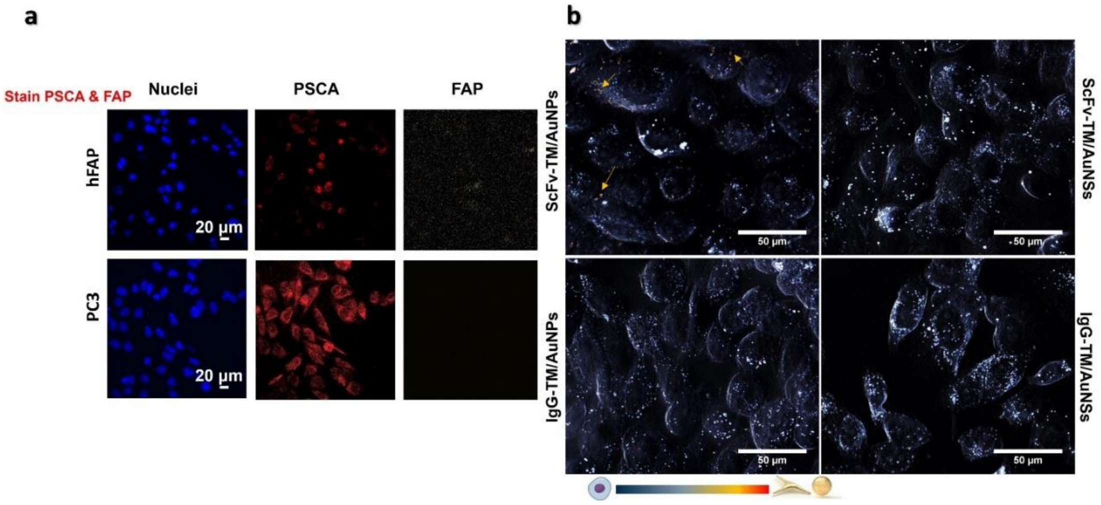

S11. (a) Immunostaining of hFAP and PC3 cell lines demonstrating FAP expression in hFAP cells. (b) PC3 cells treated with bio-nanoconjugates (0.75 OD) for 2 hours under standard incubation conditions. Minimal nanoparticle scattering (red/yellow) was observed, while blue and white scatter signals correspond to cellular organelles.
